# Unraveling the Relationship between Motor Symptoms, Affective States and Contextual Factors in Parkinson’s Disease: A Feasibility Study of the Experience Sampling Method

**DOI:** 10.1371/journal.pone.0151195

**Published:** 2016-03-10

**Authors:** Martijn P. G. Broen, Vera A. M. Marsman, Mark L. Kuijf, Robert J. Van Oostenbrugge, Jim van Os, Albert F. G. Leentjens

**Affiliations:** 1 Department of Neurology, Maastricht University Medical Center, Maastricht, the Netherlands; 2 Department of Psychiatry and Psychology, Maastricht University Medical Center, Maastricht, the Netherlands; 3 King’s College London, King’s Health Partners, Department of Psychosis Studies, Institute of Psychiatry, London, United Kingdom; Istituto Superiore di Sanità, ITALY

## Abstract

**Background:**

In Parkinson's disease (PD), the complex relationship between motor symptoms, affective states, and contextual factors remains to be elucidated. The Experience Sampling Method provides (ESM) a novel approach to this issue. Using a mobile device with a special purpose application (app), motor symptoms, affective states and contextual factors are assessed repeatedly at random moments in the flow of daily life, yielding an intensive time series of symptoms and experience. The aim of this study was to study the feasibility of this method.

**Method:**

We studied the feasibility of a five-day period of ESM in PD and its ability to objectify diurnal fluctuations in motor symptom severity and their relation with affect and contextual factors in five PD patients with motor fluctuations.

**Results:**

Participants achieved a high compliance, with 84% of assessment moments completed without disturbance of daily activities. The utility of the device was rated 8 on a 10-point scale. We were able to capture extensive diurnal fluctuations that were not revealed by routine clinical assessment. In addition, we were able to detect clinically relevant associations between motor symptoms, emotional fluctuations and contextual factors at an intra-individual level.

**Conclusions:**

ESM represents a viable and novel approach to elucidate relationships between motor symptoms, affective states and contextual factors at the level of individual subjects. ESM holds promise for clinical practice and scientific research.

## Introduction

Parkinson disease is a complex neurodegenerative disease with both motor and nonmotor symptoms. In time, most patients with Parkinson's disease (PD) eventually develop motor fluctuations such as “wearing-off” and “on-off” fluctuations. It has been estimated that two thirds of these patients also experience mood fluctuations [1, 2] and that these fluctuations are often more disabling and distressing to patients than motor symptoms [3]. Moreover, it is increasingly recognized that personal circumstances and contextual factors may also impact the severity of motor symptoms as well as the well-being of PD patients. However, the relationship between affect, motor fluctuations and their social context remains to be elucidated [4]. Traditional research methods are unlikely to provide sufficiently detailed and personal information to provide insight into the relationship between these variables. Assessing subjects several times a day during their normal daily activities, which is possible with the Experience Sampling Method (ESM), may not only provide information about the frequency and severity of emotions or motor fluctuations, but also provide valuable information on situational and behavioural moderators driving these fluctuations [5–8].

The ESM approach is gaining terrain in the study of psychopathology, [5, 6, 9, 10] and is previously used among others, in studies on depression [11], asthma [12], irritable bowel syndrome [13] and migraine [14]. To date, this approach had not yet been used in a PD population. The well known, but complex relation between the severity of motor symptoms in PD with affect and contextual factors, varying during the course of the disease, represents a particularly good target for ESM. The method assesses symptoms, contextual factors and other variables several times a day at random intervals in the subject’s natural environment [5, 6]. The primary aim of this study was to assess the feasibility of ESM in PD. In addition, we present an overview of the utility of ESM in 5 PD patients with motor fluctuations for detecting intra-individual associations between motor fluctuations, affective states and contextual factors.

## Methods

### Participants

This study was part of a clinical initiative to assess the feasibility of ESM for routine outcome monitoring in a psychiatric outpatient population [9]. The parent study was exempt from ethical approval since it concerns routine clinical follow-up measurements. For our pilot study five consecutive PD patients with motor fluctuations from the multidisciplinary movement disorder clinic were asked to participate in this initiative to assess the feasibility of the ESM method to detect intra-individual fluctuations. PD was diagnosed according to the Queens Square Brain Bank criteria [15]. Subjects with known cognitive impairment, operationalized as an MMSE <24, were excluded given anticipated difficulties in working with the mobile ESM application (PsyMate device). All approached subjects gave written informed consent. The parent study was approved by the Institutional Review Board of Maastricht University Medical Center.

### The Experience Sampling Method (ESM)

ESM is based on multiple repeated (within-subject) mini-measurements of experience (motor symptoms, anxiety, affect, wellbeing, motivation, stress) and context (medication use, stressors, situations, activities) at unselected semi-random moments in daily life. Within-subject data provide subjects and health professionals with the opportunity to follow intra-individual changes in relation to in-the-moment daily life situations and experiences. As it assesses the occurrence of motor symptoms, mental states and contexts in the flow of daily life, ESM Is a validated, structured diary technique to assess subjects in their daily living environment [5, 16]. ESM is able to follow the impact of motor and mental states, as well as their context, on each other over time.

### Measures

**Fig 1:**
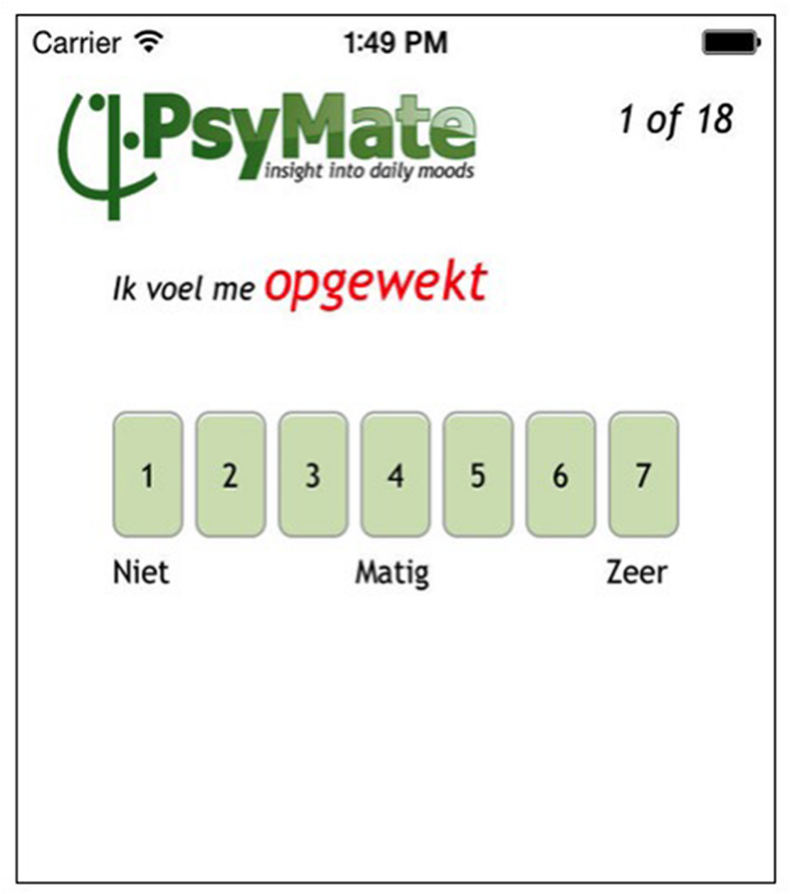
Screenshot of the question “Ik voel me opgewekt”(“I feel cheerful”) on a 7 point Likert scale. A score of 1 indicates “niet” (“not at all”), a score of 4 “matig” (“moderate”) and a score of 7 “Zeer” (“Very much so”).

The PsyMate device used in this study [17], was an iPod touch with a special ESM app installed. The device is programmed to generate 10 beeps per day at semi-random moments in 90-minutes time blocks between 7:30 and 22.30, for 5 consecutive days (total of 50 measurement points). At each beep, the PsyMate presents a number of questions on the experience of motor symptoms, affective states and contextual factors, which are recorded through a touch screen (Fig 1, screenshot question). First, subjects were asked if they felt in their “on” or “off” state, the latter being a state where in their opinion the dopaminergic medication was not working anymore (due to the short half-time of levodopa preparations). For example, when just before the next medication gift the subject experienced more motor- (return of parkinsonism), sensory- or autonomic symptoms, they answered they were in an “off” state. On the contrary, when subjects experienced few complaints during dopaminergic treatment, they answered “on”. We included 5 Parkinson-specific questions, rating the symptoms ‘tremor’, ‘rigidity’, ‘problems with walking’, ‘balance problems’ and ‘dyskinesia’. These questions were selected from the Unified Parkinson Disease Rating Scale (UPDRS) which is a commonly used scale in PD [18]. Since this was a pilot study we only included general motor symptoms and not more specific motor symptoms such as freezing, which is only present in a small proportion of PD patients. All of the included motor symptoms are strongly correlated with quality of life [19]. To assess affect state we used a constructed composite score for positive affect (PA) and negative affect (NA) [11], in line with previous studies [20, 21]. PA comprised the weighted average of scores on the affect adjectives: ‘happy’, ‘satisfied’, ‘relaxed’ and ‘feeling well’ and for NA: ‘insecure’, ‘lonely’, ‘anxious’, ‘irritated’, ‘guilty’, ‘suspicious’ and ‘threatened’. As contextual factors, the whereabouts, presence of others, and activities of the subject were registered, as well as how comfortable the subject felt in these circumstances. All PD symptoms and affect adjectives were rated by subjects on a 7-point Likert scale ranging from 1 = ‘not at all’ to 7 = ‘very’. A complete overview of the questions is given in S1A Table. The questions were the same for each measurement point. In addition, once in the morning and once in the evening, subjects had to fill in additional ‘morning’ and ‘evening’ questionnaires (S1B Table). The application uploaded the responses in an anonymized central database, from which they were analyzed. The data collection method can be considered ecologically valid measures of the subject’s circumstances and affective fluctuations in the flow of daily life [5, 6, 22].

### Assessment

Baseline demographic and disease-related characteristics were collected during a visit at the outpatient clinic (MPGB). In addition, the following scales were applied: the Hoehn and Yahr stage [23], the UPDRS to quantify motor symptoms, the Mini-Mental State Examination (MMSE) to assess global cognitive function [24], the Beck Depression Inventory II (BDI-II) to assess depressive symptoms [25], the Parkinson Anxiety Scale (PAS) [26] to assess anxiety symptoms and the Parkinson Disease Questionnaire-8 (PDQ-8) [27] to assess quality of life. During the assessment subjects received oral information on how to interpret the question about the “on” or “off” state. On the same day, information about the PsyMate as well as a demonstration was given in person (VAM). Subjects were given the option to either download and use the PsyMate on their own smartphone (iOS or Android), or to use an iPod touch provided by Maastricht University Medical Centre for the duration of the study. During the following five consecutive days the PsyMate generated a total of 50 semi-random measurement points, 10 per day. At day two, subjects were called to check whether everything was clear and functioning correctly. At any time during the study period, subjects could ask for help by calling one of the investigators (VAM). After five days, they returned the device and the data were collected in an anonymized central database. Two weeks later, the subjects were interviewed by telephone about their experiences with the device (see S1C Table for evaluation questions).

### Statistical analysis

Before running the analysis, missing items due to technical issues of the device were excluded from the data set. First, descriptive statistics were calculated for all PD symptom variables, as well as PA and NA. Since all variables were not normally distributed (kurtosis values ranging from 2.9 to 28.7), correlations were computed with Spearman’s rho. Strength of the correlation was defined as *r* < 0.3 very small, *r =* 0.3–0.5 small, *r* = 0.5–0.7 moderate and *r* > 0.7 strong. Additionally, effect sizes were calculated by calculating Cohen’s *d*, e.g. a 1 point change on the scale has an effect size of 1/standard deviation of the variable. Strength of the effect size was defined as medium *d = 0*.*3–0*.*6* and large *d > 0*.*6*. Analyses were done using the statistical software program STATA (version 13.1 for Mac).

## Results

Table 1 shows baseline characteristics of the 5 subjects. The mean age was 60.4yr (SD 6.1). All subjects had a Hoehn and Yahr stage of 2–2.5, which indicates bilateral or midline involvement of the disease, without impairment of balance or a recovery on the pull test. The mean UPDRS III score was 22 (SD 6.2). On the PAS, subject 1 and 4 scored above the cut-off point of 13/14 indicating clinically relevant anxiety. Subject 4 also scored above the cut off score of 14/15 on the BDI-II, which fits with a mild depression. Quality of life of the subjects was relatively good with a maximum score of 13 out of 32, with higher scores indicating a lesser quality of life.

**Table 1:** Baseline characteristics of the 5 subjects.

	Sex	Age (yrs)	Disease duration (yrs)	Marital status	Education	Side of onset	Tremor	LEDD	UPDRS III (range 0–108)	HY (range 1–5)	MMSE (range 0–30)	PDQ-8 (range 0–32)	PAS(range 0–48)	BDI-II (range 0–63)
**1**	M	65	6	Single	HE	Left	Yes	1100	22	2,5	30	12	21	11
2	M	63	8	Married	VE	Right	No	1306	15	2	29	7	11	1
3	M	50	5	Single	VE	Right	No	557	25	2	29	9	11	8
**4**	V	60	11	Married	VE	Right	Yes	835	32	2	30	13	22	16
5	M	64	10	Married	U	Right	No	1242	16	2	29	10	9	3

Abbreviations: HE, higher professional education; VE, vocational education; U, university; LEDD, Levodopa equivalent daily dose; UPDRS III, Unified Parkinson Disease Rating Scale part III; HY, Hoehn and Yahr scale; M;MSE, Mini Mental State Exam; PDQ-8, Parkinson’s Disease Questionnaire; PAS, Panic and Agoraphobia Scale; BDI II, Beck Depression Inventory.

### Compliance

A mean total of 84% (range 76–96%) of all 50 measurement points were completed correctly. Previous research has shown that ESM data validity is compromised if less than one third of beeps yield data [5]. All subjects in met this requirement. Subject 2 and 3 both missed three beeps because they did not hear the sound of the alarm and subject 4 went swimming several times and missed four beeps when she was in the water. Unfortunately, when transferring data from the device of subject 1, data of fifteen measurement points were lost due to connection malfunction. The exact cause of this malfunction remains unclear. There was no significant difference in terms of compliance between the three subjects using their own smartphone and the two using an Ipod (89% vs 83%).

### User friendliness, evaluation and feedback

All subjects found the information about the PsyMate device clear and concise. None of them said they changed their daily behaviour. This occasionally led to a missed assessment point when they were showering, swimming, riding a bike or driving a car. Three subjects used their own smartphone and two used an iPod. These two were not inconvenienced by having to carry the iPod device with them. Beep assessments never took more than 5 minutes, with a mean completion time between 2 and 3 minutes. The mean utility score that the subjects gave the device was 8 on a 10-point scale. Some subjects suggested also including questions about exercise, since in their own experience this also influences their affect. Three subjects mentioned that repetitive assessments tended to get boring. However, studying the relationship between affect and motor fluctuations and contextual factors was considered very meaningful by all subjects and they all would participate in future ESM studies.

### Ability to detect diurnal fluctuations in motor symptom severity at an individual level

**Fig 2:**
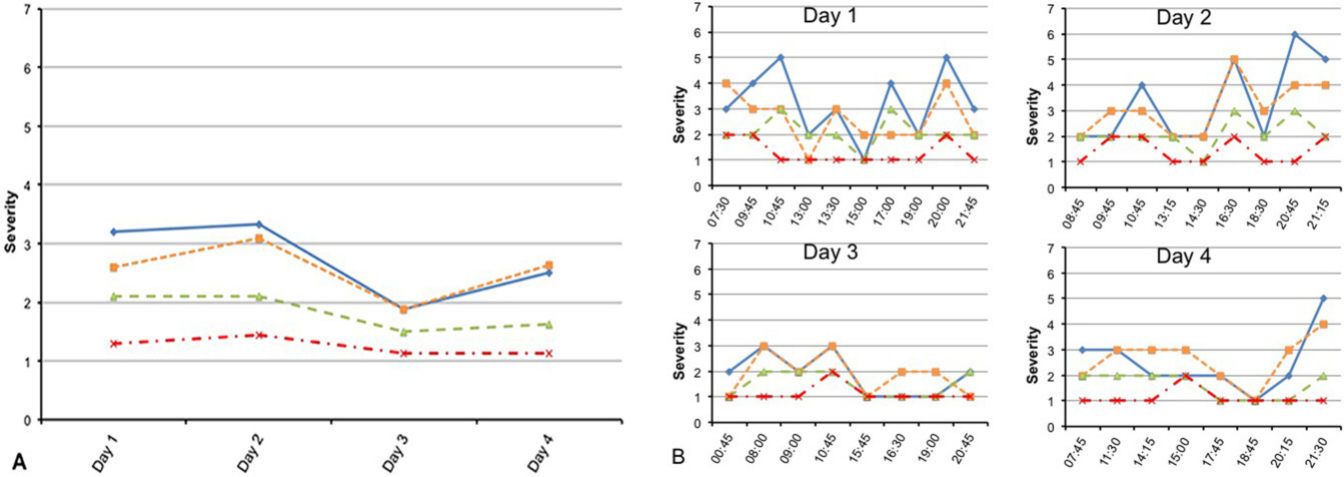
**a and b.** Cross-sectional overall daily assessment (Fig 2a) versus real-time measurements (Fig 2b) of motor symptoms in subject 4. Legend: Y-axis: item score (1 = not all, 7 = very much); X-axis: time. Blue solid line: Tremor; Orange line: Dyskinesia; Red line: Trouble Walking; Green line: Rigidity.

Fig 2 shows the overall motor symptom severity and the real-time diurnal fluctuations in motor symptom severity of subject 4. Cohen’s *d* for tremor was .75 and 1.28 for PA, with indicates a large, meaningful effect size of a 1 point change on the Likert scale. Similar patterns were found in all subjects. These figures show that ESM is sensitive enough to capture clinically relevant diurnal fluctuations of motor symptom severity.

### Ability to detect intra-individual relationships between motor symptoms, affect and contextual factors in 5 PD subjects with motor fluctuations

**Fig 3:**
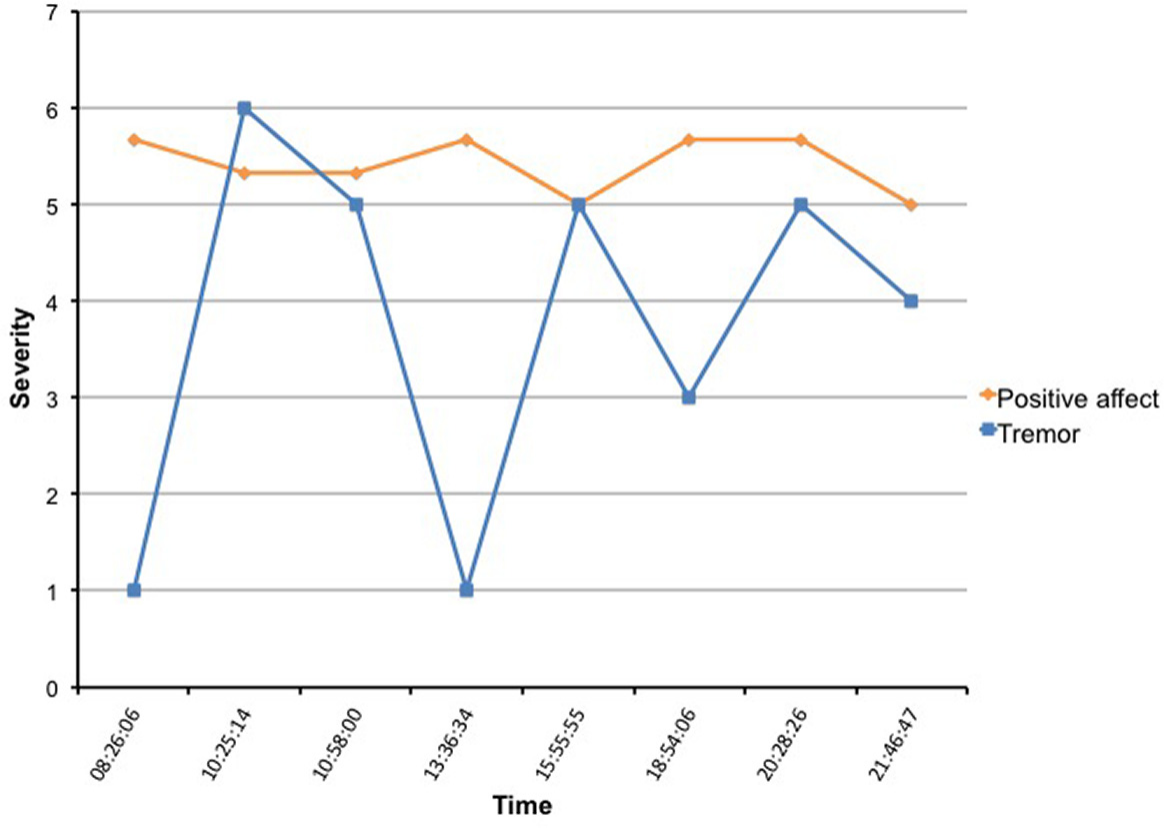
Example of real time assessment of tremor and positive affect in subject 1.

**Fig 4:**
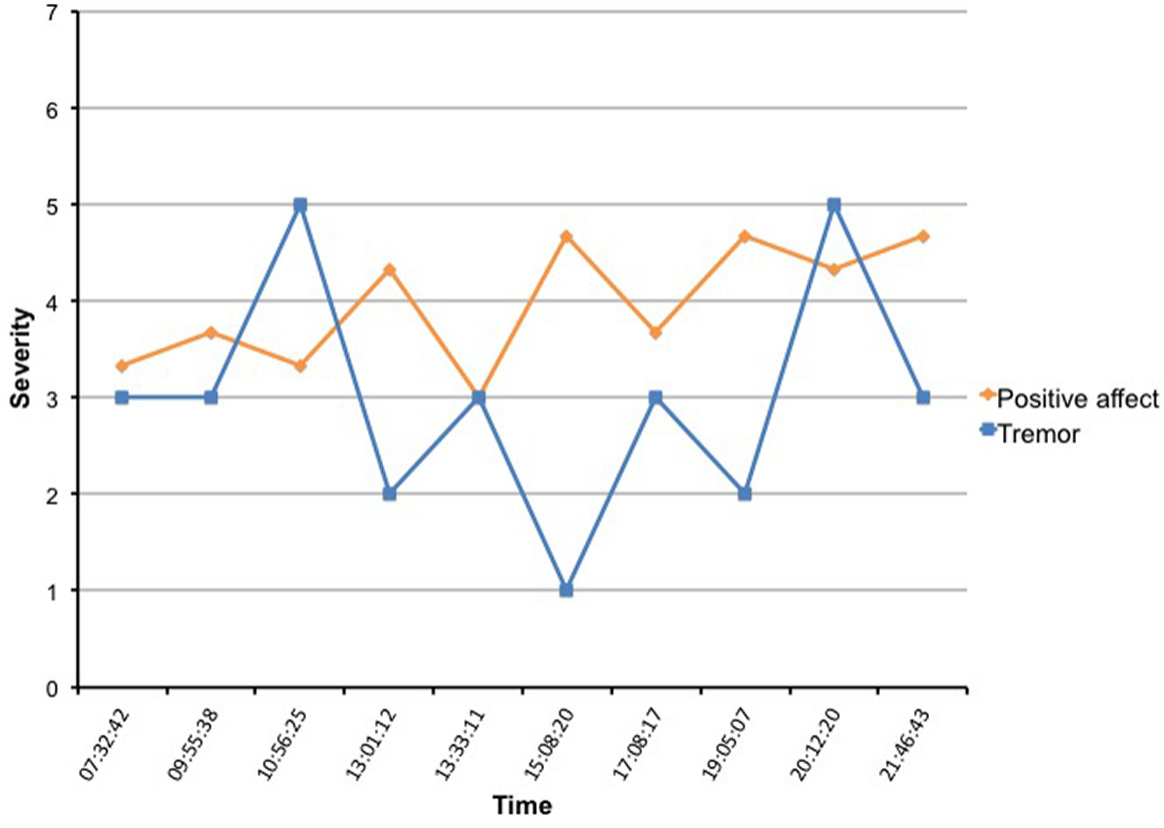
Example of real time assessment of tremor and positive affect in subject 4.

First, since subjects with mood fluctuations are more likely to have higher scores on psychiatric rating scales [28], we expected that subjects 1 and 4, which had the highest scores on the anxiety and depression rating scales, would also have the strongest correlation between motor symptoms, affect and possibly contextual factors. This was especially true for the severity of the tremor; both subject 1 and 4 had a moderate negative correlation between positive affect and the severity of the tremor (*r* = -0.5, *p <* .01). For subject 1, Cohen’s *d* for tremor was .57 and 2.63 for PA, indicating a large effect size for a 1-point change on the Likert scale. See Figs 3 and 4 for an example of the fluctuations of tremor and affect during the day. In Fig 3, one can see that the severity of the tremor fluctuates heavy, but the positive affect line shows only minor changes. This is most likely because the PA is a weighted average of 4 affect adjectives and when looking closer at the data it turned out that mainly the variable “satisfied” had a correlation with tremor (*r* = -0.6, *p* < .01). In subject 4, feeling relaxed (*r* = -0.6, *p* < .01), satisfied (*r* = -0.6, *p* < .01) and feeling happy (*r* = -0.4, *p* < .01) all had a negative correlation with tremor severity, visible as a more fluctuating PA pattern in the diagram. On the contrary, subject 5 had the lowest scores on the rating scales and we were unable to find a significant correlation between affect and any of the motor or contextual factors.

**Fig 5:**
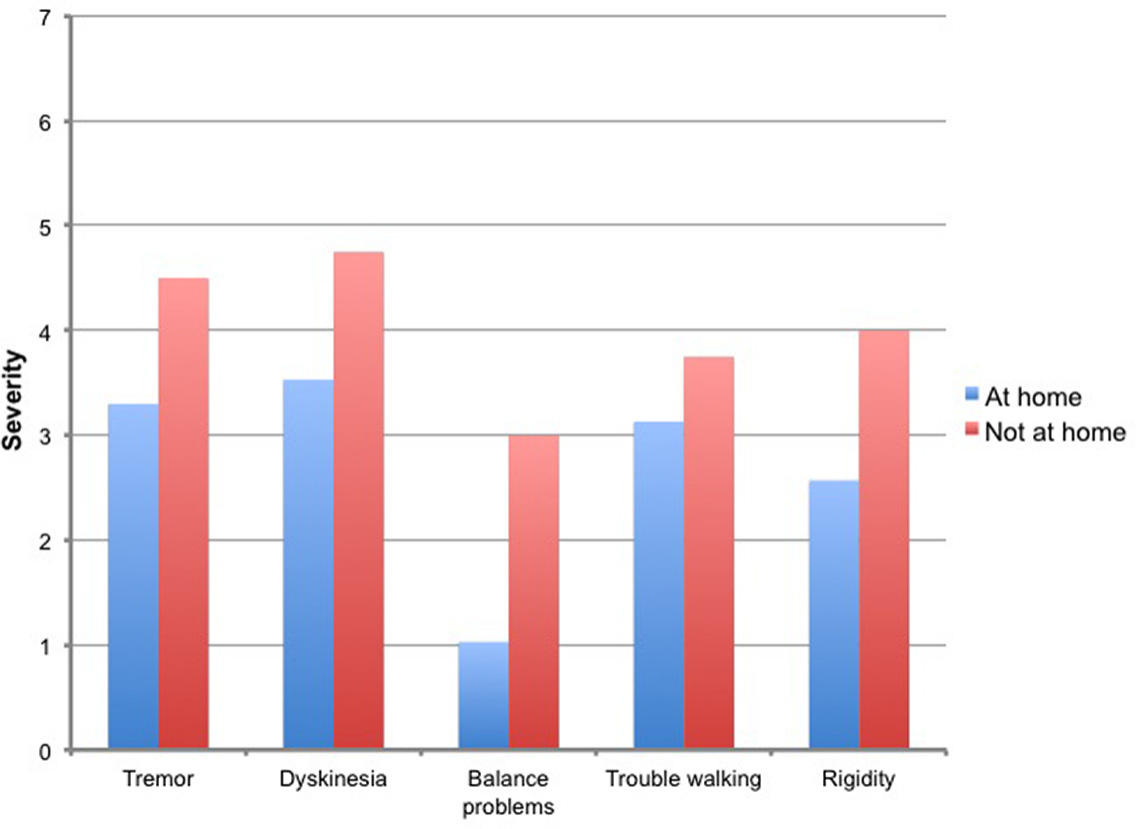
Clustered column diagram of motor symptom severity in subject 1. Legend: Y-axis: item score (1 = not all, 7 = very much); X-axis: motor symptoms.

Second, we evaluated the influence of context on the severity of motor symptoms. Again, in subject 1, the effect was most impressive. Fig 5 shows that all motor symptoms were more severe when he was not at home but in public. In the other subjects it was difficult to find a correlation between contextual factors and symptom severity, mainly because the measurement point were not evenly distributed, e.g. most of the measurements were taken at home, or in company with their spouses with only a few measurements taken in the opposite situation which makes a statistical comparison difficult.

Third, to test whether individual motor symptoms were logically correlated, e.g. improved rigidity will normally lead to impaired balance or walking difficulties, we calculated these correlations in subject 3, who had a moderate score on the UPDRS III. We found a strong correlation between rigidity and the severity of walking difficulties (*r* = 0.8, *p* < .01). Balance problems were moderate correlated with dyskinesia (*r* = 0.6, *p* < .01) and also strongly correlated with walking difficulties (*r* = 0.8, *p* < .01). For subject 3 Cohen’s *d* for balance problems was 1.90, for walking difficulties 1.26 and 3.51 for dyskinesia. Similar correlation coefficients were found in the other subjects, which indicated that their symptoms were logically scored.

Last, we tried to detect effects of dopaminergic treatment on the motor symptoms or affect fluctuations. However, in this pilot application subjects were not asked to mark at what exact time they took their medication, so we were only able to visible link their prescription time with the severity of their symptoms. For example, according to her prescription time subject 4 took levodopa medication at 7 and 11 o’clock in the morning, three o’clock in the afternoon and 7 and ten o’clock in the evening. Looking at her severity of the tremor (Fig 4), it seems that the tremor was worse just before she took her medication (point 3, 10:56) and less severe just after her medication (point 6, 15:08). Since the tremor severity and positive affect were correlated, this was also true for her affective state, having a more positive affect just after the medication gift. However, we realize this is not very accurate, since actual medication times can differ notoriously from prescription times.

## Discussion

This pilot study shows that ESM is a viable and promising method for evaluating affect and motor fluctuations in relation to contextual factors in PD. Subjects achieved a high compliance rate without disturbance of normal daily activities. The average rating of the utility of the device was an 8 on a 10-point scale. Subjects were confident that ESM had the potential of revealing meaningful information of factors associated with fluctuations in motor symptom severity. ESM proved to be sensitive enough to capture extensive diurnal fluctuations in motor symptom severity, affective states and contextual factors.

The advantage of ESM is that assessments take place in the flow of daily life, in the natural environment of the subject. This is important since contextual factors play an important role in motor symptom severity, affect and behavior [29, 30]. By assessing subjects during normal activities in their natural environments, ESM avoids some of the methodological problems commonly encountered in research, which has implications for treatment. First, it reduces recall bias. Current assessments in clinical settings often rely on retrospective questionnaires and this possible leads to several sources of recall bias. It has been shown in depressed subjects that there is a high discrepancy between retrospective ratings and actual experiences [31]. Another advantage is that by using an electronic device, investigators can be confident that the ratings were actually completed at the time specified by the research design, which one cannot be sure of when using repeated paper questionnaires. This avoids the problem of “back-filling” the diaries when subjects neglect to make ratings at the scheduled time. A clinical advantage of the ESM approach is that multiple repeated assessments within each subject, forming an intensive time series of symptoms, context and experience, increases the power of the data to such an extent that analysis of single subject time series becomes possible. This enables the recognition of highly individual patterns of reactivity to contextual factors, and may facilitate the prediction of ‘off’-periods and other important transitions [32]. Person-specific patterns may be fed back to the subject to enhance insight into factors associated with symptom severity and facilitate self-management. Behavioural insight may enhance feelings of mastery over the symptoms, and facilitate coping or adjustment of activities. Providing feedback may also lead to an increased sense of involvement in treatment, better adherence and better treatment outcome, as was previously reported in other subject populations, such as subjects with depression, migraine and asthma [33–35].

The monitoring of treatment progress is also a promising clinical application. ESM may reveal relatively small treatment effects by avoiding clouding due to recall bias when using retrospective self-report measures [36]. This would allow for more targeted and individualized pharmacotherapy. A practical advantage is that ESM software is freeware and can be downloaded for free as an app on smart phones or iPods.

A possible limitation of ESM used in our study is that it needs a certain level of motor function to use the telephone device but also a sufficient cognitive level to understand the information and learn to use the PsyMate program. For this reason, the use of ESM is limited to a selected population with sufficient cognitive and motor function, which in future studies could lead to selection and lack of generalizability. However, in most PD studies a certain level of cognitive function is already required. Second, the presence of motor fluctuations was based on self-reports, i.e. were not observer-rated, which may differ objective ratings of fluctuations. Previous studies on motor and mood fluctuations reported a discrepancy between self-reported and physician-documented motor fluctuations, which could be due to “over reporting” of fluctuations by subjects but also by the tendency of neurologists to continue to view subjects who have developed motor fluctuations as “fluctuators”, regardless of how well controlled their symptoms are at a given time [28]. Such overestimation of fluctuations could be a problem, especially when treatment modifications are made due to ESM results. To further objectify the motor fluctuations additional use of an accelerometer is a possible solution, a method that is currently finding its way in studies of movement and gait disorders in PD [37, 38]. With respect to the overestimation of emotional fluctuations, previous research has shown that self-reports are an useful source of data when dealing with immediate experiences such as affective states [16, 39]. In our study it seems that the self-reports on emotional items are plausible representations of reality. However, this needs to be validated further. Last, our study was part of a larger study on ESM in a psychiatric population. Further studies are needed to validate our preliminary findings on the ability of ESM to detect meaningful intra-individual relationships. These future studies should implement an ESM questionnaire optimized for PD patients, e.g. more questions about motor symptoms and a more precise medication time queue which enables researches to better detect medication influences. In addition, PD patients with different stages of the disease should be included, to extend our findings in subjects who are more disabled. However, taken all these limitations into account, we think that ESM is a feasible method in PD and holds promises for future scientific and clinical implications.

## Conclusion

ESM may be a viable and useful method in PD. Subjects reached a high compliance rate and were able to rate multiple symptoms simultaneously in a brief period of time, without disturbance of daily activities. The study shows that ESM technology is sensitive enough to capture extensive diurnal fluctuations of motor symptom severity, affective states and contextual factors in individual subjects. Therefore ESM could be a useful tool for fine grained analyses of how these variables impact each other in the flow of daily life in individual subjects, and how these patterns may be altered as a function of treatment. Detailed feedback to the subject may enhance insight and facilitate self-management, shared decision making and compliance.

## Supporting Information

S1 Table A) Experience Sampling Methods protocol, question per beep. B) Additional questions once in the evening and once in the morning, C) Evaluation questions asked during telephone interview.
(DOCX)
