# Supplementary material for: Unraveling the Relationship between Motor Symptoms, Affective States and Contextual Factors in Parkinson’s Disease: A Feasibility Study of the Experience Sampling Method
Source: PLoS One. 2016 Mar 10;11(3):e0151195. doi: 10.1371/journal.pone.0151195 (PMC4786263; doi:10.1371/journal.pone.0151195)
Supplement: S1 Table — A) Experience Sampling Methods protocol, question per beep. B) Additional questions once in the evening and once in the morning, C) Evaluation questions asked during telephone interview. (DOCX) [file pone.0151195.s001.docx]

**SUPPLEMENTARY DATA**

**S1A table. Experience Sampling Methods protocol, question per beep.**

| **Domain** | **Item** | **Description** | **Score** |
| --- | --- | --- | --- |
| **Mood** | 1 | I feel happy | 1-7 (1=not, 4=moderate, 7= very) |
|  | 2 | I feel insecure | 1-7 |
|  | 3 | I feel relaxed | 1-7 |
|  | 4 | I feel irritated | 1-7 |
|  | 5 | I feel satisfied | 1-7 |
|  | 6 | I feel lonely | 1-7 |
|  | 7 | I feel afraid | 1-7 |
|  | 8 | I feel down | 1-7 |
|  | 9 | I feel guilty | 1-7 |
|  | 10 | I rack my brain | 1-7 |
|  | 11 | I feel suspicious | 1-7 |
|  | 12 | I feel threatened | 1-7 |
|  | 13 | Currently I am: | 1=”on”, 0=”off” |
| **Parkinson** | 14 | I experience tremor | 1-7 |
|  | 15 | I experience rigidity | 1-7 |
|  | 16 | Walking is difficult | 1-7 |
|  | 17 | I experience balance problems | 1-7 |
|  | 18 | I experience dyskinesia | 1-7 |
|  | 19 | Personal complaints: | Open question |
|  | 20 | In general, I feel well | 1-7 |
| **Context** | 21 | Currently I am: | Resting/working/housework/hygiene/  eating,drinking/relaxing/conversating/other |
|  | 22 | I rather do something else | 1-7 |
|  | 23 | Where am I? | Home/work/ friends place/public place/on the go |
|  | 24 | With who am I? | Partner/family/friends/collegues/nobody |
|  | 25 | I find this pleasant | 1-7 |
| **Somatic** | 26 | I am hungry | 1-7 |
|  | 27 | Since the last beep I used: | Nothing/caffeine/nicotine/alcohol/medication/cannabis/  food/other |
|  | 28 | I am tired | 1-7 |
|  | 29 | I am not feeling well | 1-7 |
|  | 30 | I have pain | 1-7 |
|  | 31 | I experience: | Headache/abdominal pain/breathing difficulties/muscle ache/ obstipation/tinnitus/dizziness/ dyspnea/ palpitations/incontinence |
| **Events** | 32 | The most important event since the last beep was: | Open question |
|  | 33a | The event was: | -3 = very displeasing, 0 = neutral , 3 = very pleasant |
|  | 33b | The event was: | -3 = not important, 0 = neutral, 3 = very important |
|  | 33c | The event was under my control | 1-7 |
|  | 33d | I expected this event | 1-7 |
|  | 34 | This beep was unpleasant | 1-7 |

**S1B Table. Additional questions once in the evening and once in the morning.**

| **Item** | **Scale** |
| --- | --- |
| **EVENING** | |
| In general, I felt well today | 1-7 (1=not, 4=moderate, 7= very) |
| In general, I felt tired today | 1-7 |
| In general, I felt tense today | 1-7 |
| In general, I puzzled over a lot today | 1-7 |
| Today, I experienced Parkinson symptoms | 1-7 |
| Today, I experienced headache | 1-7 |
| Today, I experienced stomachache | 1-7 |
| Today, I had difficulties breathing | 1-7 |
| Today, I had muscle pain | 1-7 |
| Today, I experienced obstipation | 1-7 |
| Today, I experienced tinnitus | 1-7 |
| Today, I experienced palpitations | 1-7 |
| I was dizzy today | 1-7 |
| I had dyspnea today | 1-7 |
| I experienced micturition problems | 1-7 |
| Filling in these questions influenced my mood | 1-7 |
| Without PsyMate, I would have done different things today | 1-7 |
| **MORNING** | |
| How long did it take to fell asleep yesterday? | 0-5min/5-15min/15-30min/30-45min/45min-1h/ 1-2h/2-4h/>4h |
| How many times did I wake up last night? | 0- >5 times |
| How long was I awake this morning, before I got up? | 0-5min/ 5-15min/15-30min/30-45min/45min-1h/ 1-2h/2-4h/ >4h |
| I slept well | 1-7 |
| I feel fit | 1-7 |

**S1C Table. Evaluation questions asked during telephone interview**

1. Was the goal of our study clear to you?

2. Was the information about the study clear and sufficient for you?

3. Were all the question clear? If not, which questions were not clear?

4. Did you change your daily behavior during the study period? If so, why?

5. Did you bother caring around the PsyMate device (or cell phone) all day long?

6. Did you miss any measurement points? If so, how many and why (for example: I did not hear the beep/forgotten/embarrassment)?

7. Did you fill in the questions by yourself or with your partner?

8. Please give an overall score for the utility of the device and program (0-10)?

9. How long did it take to fill in one measurement point (0-1min, 1-2min, 2-5min, >5min)?

10. Do you have any suggestions or comments to improve the study design?
